# Spatial Transcriptomics: Technical Aspects of Recent Developments and Their Applications in Neuroscience and Cancer Research

**DOI:** 10.1002/advs.202206939

**Published:** 2023-04-07

**Authors:** Han‐Eol Park, Song Hyun Jo, Rosalind H. Lee, Christian P. Macks, Taeyun Ku, Jihwan Park, Chung Whan Lee, Junho K. Hur, Chang Ho Sohn

**Affiliations:** ^1^ Center for Nanomedicine Institute for Basic Science Yonsei University Seoul 03722 Republic of Korea; ^2^ Graduate Program in Nanobiomedical Engineering Advanced Science Institute Yonsei University Seoul 03722 Republic of Korea; ^3^ School of Biological Sciences Seoul National University Seoul 08826 Republic of Korea; ^4^ Graduate School of Medical Science and Engineering Korea Advanced Institute of Science and Technology (KAIST) Daejeon 34141 Republic of Korea; ^5^ School of Life Sciences Gwangju Institute of Science and Technology (GIST) Gwangju 61005 Republic of Korea; ^6^ Department of Chemistry Gachon University Seongnam Gyeonggi‐do 13120 Republic of Korea; ^7^ Department of Genetics College of Medicine Hanyang University Seoul 04763 Republic of Korea

**Keywords:** bioinformatics, cancer, neuroscience, RNA, spatial transcriptomics

## Abstract

Spatial transcriptomics is a newly emerging field that enables high‐throughput investigation of the spatial localization of transcripts and related analyses in various applications for biological systems. By transitioning from conventional biological studies to “in situ” biology, spatial transcriptomics can provide transcriptome‐scale spatial information. Currently, the ability to simultaneously characterize gene expression profiles of cells and relevant cellular environment is a paradigm shift for biological studies. In this review, recent progress in spatial transcriptomics and its applications in neuroscience and cancer studies are highlighted. Technical aspects of existing technologies and future directions of new developments (as of March 2023), computational analysis of spatial transcriptome data, application notes in neuroscience and cancer studies, and discussions regarding future directions of spatial multi‐omics and their expanding roles in biomedical applications are emphasized.

## Graphical abstract

Spatial transcriptomics enables high‐throughput investigation of spatial localization of transcripts and related analyses in various applications for biological systems. This review provides a directed overview of the rapidly evolving field of spatial transcriptomics emphasizing technical insights, applications, and future directions in the fields of neuroscience, cancer studies, and translational biomedicine.

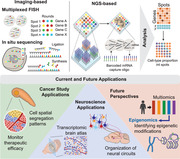

## 1 Introduction

### 1.1 Spatial Transcriptomics: Emerging Technology

Each cell in a multicellular organism interacts with the surrounding environment. Stem cells differentiate during development primarily through cell‐to‐cell interactions and subsequent signaling, which is governed by the relative positions of cells within the embryo.^[^ ^1^, ^2^ ^]^ The spatial organization of tissues regulates the expression of transcription factors related to differentiation and ultimately generates a robust organization of cellular structures related to their functions.^[^ ^3^, ^4^, ^5^ ^]^ Another example that illustrates the importance of spatial organization is cancer tissue, in which cells actively interact with the surrounding tumor microenvironment to generate suppressive conditions that block the action of immune cells, thereby bypassing immune defense mechanisms and facilitating proliferation.^[^ ^5^, ^6^ ^]^ To understand the complexity of biological systems ranging from various physiological phenomena to the pathological principles of diseases, it is necessary to assess the functions of individual cells and their interactions to orchestrate complex functions of tissues and organs. Strategies for examining these biological principles include exploring cells that exist in the tissue (cell‐type inventory) and their spatial arrangement and interaction with each other (understanding their spatial organization).

Molecular expression patterns of diverse biological states have been analyzed by RNA sequencing owing to the efficient and sensitive detection of RNA by the simple amplification of nucleic acids by polymerase chain reaction (PCR) and high‐throughput readouts by next‐generation sequencing (NGS). RNA sequencing has served as one of the major approaches for basic biological research and clinical diagnosis because the transcriptome serves as a blueprint of the proteome, the actual functional proteins of the cell, and therefore reflects the current cellular state.^[^ ^7^ ^]^ Furthermore, recent advances in single‐cell transcriptome analysis techniques allow researchers to analyze transcriptomes with unprecedentedly high throughput and single‐cell resolution.^[^ ^8^, ^9^ ^]^ Despite the improvements in sequencing technologies, single‐cell transcriptome analysis techniques are still limited in that all information related to the spatial organization of cells in the tissue is permanently lost owing to tissue dissociation.

Spatial transcriptomics provides information on the spatial distribution of gene expression profiles, thereby elucidating interesting features previously not revealed by single‐cell RNA sequencing methods that lack spatial information. Several high‐quality review papers already exist on spatial transcriptomics.^[^ ^10^, ^11^, ^12^, ^13^, ^14^ ^]^ Therefore, in this review article, we instead focus on providing a comprehensive review of the technical differences between sequencing‐ and imaging‐based methodologies, recent advances in the spatial approach utilizing spatial data and their computational interpretation, and current research and future perspectives in various fields of application in biological studies, including neuroscience and cancer, and in biomedical and clinical studies of disease.

## 2 Imaging and Sequencing‐based Spatial Transcriptomics Methods

### 2.1 Imaging‐Based Methods [Fluorescence In Situ Hybridization and In Situ Sequencing‐Based Methods]

To detect transcripts from cells, pioneering studies reported single‐molecule fluorescence in situ hybridization (smFISH) methods that employed in situ hybridization of reverse‐complementary oligo probes conjugated with fluorophores.^[^ ^15^, ^16^ ^]^ These smFISH methods facilitated the detection of target RNAs with high specificity and sensitivity at the single‐molecule level. smFISH also provides single‐cell and subcellular resolution with optimized protocols for cell cultures and thin (<20 µm) tissues respectively. Recently, spatial applications of smFISH have demanded more scalable platforms in order to investigate biologically meaningful dimensions according to their detection targets.^[^ ^17^ ^]^ Fast cycling of probe hybridization allows the spatial investigation of relatively large areas with high specificity and sensitivity when targeting 20–40 highly expressed marker genes (ouroboros smFISH, or osmFISH) without barcoding.^[^ ^18^ ^]^ However, the limited multiplexing capacity of smFISH, caused by the spectral overlap of fluorescent dyes, precludes efficient transcriptome‐level spatial investigations when creating a profile of the transcriptional status of cells in a tissue.

**Figure 1:**
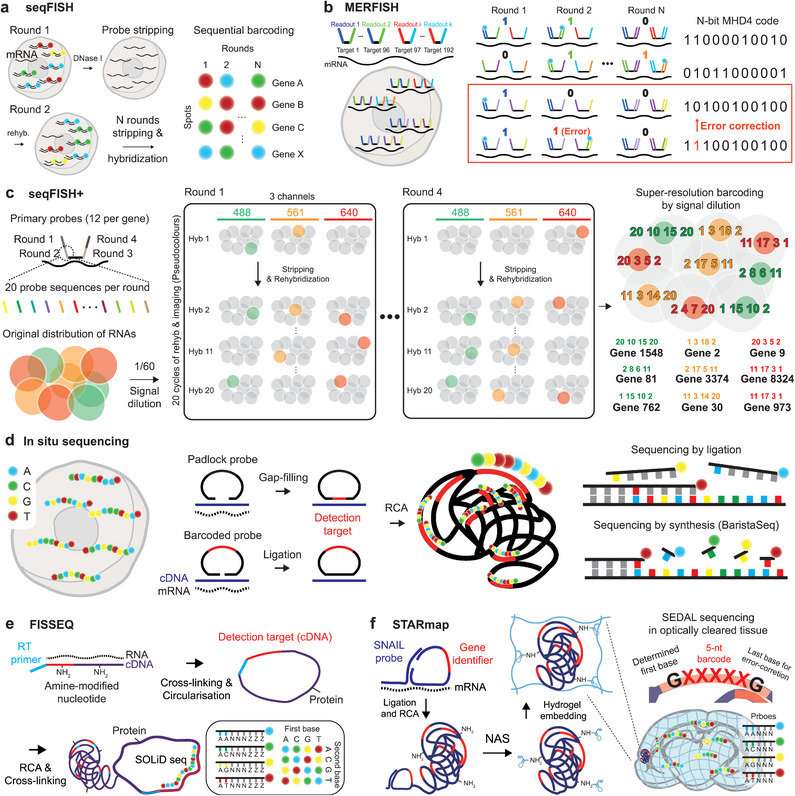
Imaging‐based spatial transcriptomics methods. a) seqFISH by DNaseI‐based digestion and sequential staining/imaging cycles to decode transcripts in space. b) MERFISH employing error correction in barcode assignment for robust barcode calling in noisy FISH‐based images. c) seqFISH+ for genome‐scale transcriptome investigation by dilution of fluorescent signals, separating individual transcripts into fluorescent spectra, and employing 20 probes per each encoding round. d) in situ sequencing methods by sequencing by ligation (ISS, FISSEQ, STARmap) and sequencing by synthesis (BaristaSeq). e) SOLiD sequencing of cDNA sequence by FISSEQ while cross‐linking cDNA and amplicon generated by rolling‐circle amplification (RCA) to adjacent proteins. f) STARmap with SNAIL probes and SEDAL sequencing for identifying gene‐specific identifiers. The polymerization of amplicons with acrylamide moieties introduced by *N*‐acryloxysuccinimide (NAS) into the hydrogel network and optical clearing by hydrogel‐histochemistry enables spatial transcriptome detection in thick tissues.

Separating the fluorescence signals obtained from individual transcripts is the key to highly increasing the multiplexing capacity. This idea has been previously implemented for DNA, but in most cases, these attempts were close to the preliminary, proof‐of‐concept stage.^[^ ^19^ ^]^ Initial efforts for multiplexed RNA profiling focused on improving the barcoding capacity using 1) spectral barcoding by super‐resolution microscopy^[^ ^20^ ^]^ and 2) sequential barcoding using DNase I (**Figure** **1**a).^[^ ^21^ ^]^ Without barcoding, different RNAs can be resolved near diffraction‐limited spots; however, the barcoding capacity can be further extended if one can resolve overlapping signals. Lubeck et al.^[^ ^20^ ^]^ first demonstrated a multiplexed barcoding scheme for resolving the RNA expression of 32 target genes in a single yeast cell by super‐resolution microscopy. This approach, however, requires expert‐level knowledge of optics for accurate implementation due to the technical difficulties of its setup. Moreover, the super‐resolution version of the spectral barcoding method consumes a very long time for imaging (i.e., a yeast cell takes 30 min to barcode 32 genes), precluding the scalable application of this method to real‐world samples. An improved protocol for a sequential barcoding scheme by DNase I dramatically enhanced the performance of repeated probing for multiplexed RNA FISH and simplified the experimental procedure so that multiplexed barcoding experiments could be executed with an ordinary epifluorescence microscope.^[^ ^21^ ^]^ Although the initial report on the DNase I‐based sequential barcoding scheme only presented its application to yeast cells, the concept of multi‐round imaging and registered barcode calling for smFISH‐based experiments was continuously adapted to the later versions of highly multiplexed FISH experiments.

In 2015 and 2016, continued efforts to develop highly multiplexed FISH methods increased the number of detected genes from a dozen to several hundred and then up to one thousand transcripts per cell [multiplexed error‐robust FISH (MERFISH)^[^ ^22^ ^]^ and sequential FISH (seqFISH),^[^ ^23^ ^]^ Figure 1a,b]. To encode as many transcripts as possible, sequential barcode schemes were implemented using repeated 3–4 color imaging cycling platforms. The key to successful and highly multiplexed barcoding is to seek schemes for improving barcode calling rates to distinguish probe signals from noise‐ and crosstalk‐prone raw images and to minimize unwanted dye quenching during fast imaging cycles. However, the barcoding schemes for ever‐increasing multiplexity are not always compatible with traditional highly expressed marker genes because these high‐copy genes easily saturate the fluorescence field of view, resulting in poor barcode calling rates due to signal overlap.^[^ ^24^, ^25^ ^]^ To evenly distribute the “loading” of gene expression in each fluorescence channel, careful estimation of expression from bulk sequencing results is employed to design orders of imaging and to determine which genes can be co‐imaged. Super‐resolution imaging by localization microscopy was employed to resolve more transcripts in each diffraction‐limited spot, which finally enabled true transcriptome‐level detection in the order of ten thousand transcripts per cell (seqFISH+) (Figure 1c).^[^ ^26^ ^]^ Error‐robust barcoding schemes, corrections for optical aberrations, lowering autofluorescence by tissue clearing, and semi‐automated microfluidics‐based staining and imaging cycling systems further improved the quality of the resulting datasets in comparison to those obtained by sequencing‐based methodologies (discussed in the next section) (Figure 1b).^[^ ^27^, ^28^ ^]^ From the point of view of biomedical and translational/clinical applications, knowing whether a particular method can cover clinically meaningful ranges of tissue size and thickness is critical. Although these multiplexed FISH‐based methods have excellent sensitivity and coverage of transcriptomes with subcellular resolutions, they all suffer from substantially long imaging times, which limits practical tissue size and thickness. For example, in seqFISH+, an imaging time of 1 week is required to image a single optical plain of a partial region of the cortex in a thin coronal tissue section of the mouse brain when performing 80 rounds of hybridization/imaging to detect 10 000 transcripts. Even after this effort, a typical tissue section in these methods is only 10–20‐µm thick, and therefore, the cells at this thickness are mostly not intact. In addition to the difficulty in registration and barcode calling from these large raw image datasets, the resulting data suffer from incomplete analysis of single‐cell expression profiles that are inherently non‐intact in a spatial context. Future efforts should focus on developing fast imaging schemes by implementing simple and easy signal amplification, which will eventually enable the analysis of thick tissues by improving sample coverage. Easy and user‐friendly interfaces in data acquisition and analysis for commercialization processes are crucial for facilitating widespread usage. Enhanced Electric FISH (EEL FISH) is an electrophoresis‐aided large tissue RNA sampling and multiplexed FISH study that attempts to reduce data acquisition time by transferring RNA from 10‐µm‐thick tissue to the plane of an RNA capture slide for imaging the transcriptome of thick tissues with an epifluorescence microscope.^[^ ^29^ ^]^

In contrast to sequential imaging of barcoded FISH probes, in situ sequencing (ISS) is an alternative approach for identifying a larger number of RNA‐targeting probes by direct imaging of nucleotide sequences in situ (Figure 1d–f). In principle, both FISH and ISS provide similar transcriptome information at subcellular resolution. However, ISS can read nucleotide sequences directly from tissues, which is a critical feature that allows the possibility for new applications such as in situ detection of single nucleotide polymorphisms. In 2013, the first ISS study employed sequencing‐by‐ligation chemistry to read short sequences of gene barcodes in situ.^[^ ^30^ ^]^ In ISS, reverse‐transcribed complementary deoxyribonucleic acids (cDNAs) are hybridized with padlock probes containing gene‐specific barcode sequences, and the padlock probe is ligated at the location of specific hybridization before being amplified by rolling‐circle amplification (RCA) using a circularized padlock primer probe (Figure 1d). Sequential imaging by sequencing‐by‐ligation allows the identification of repeatedly amplified barcode sequences in situ. Fluorescentin situ sequencing (FISSEQ) detects RNA by employing sequencing by oligonucleotide ligation and detection (SOLiD) chemistry to directly read cDNA sequences synthesized by random hexamers and provide an unbiased examination of the whole transcriptome distribution (Figure 1e).^[^ ^31^, ^32^ ^]^Spatially‐resolved transcript amplicon readout mapping (STARmap) uses a novel in situ sequencing chemistry called sequencing with error‐reduction by dynamic annealing and ligation (SEDAL) for the highly efficient synthesis of barcoded probe sequences (Figure 1f).^[^ ^33^ ^]^ For volumetric investigations of in situ RNA distribution, STARmap uses CLARITY‐based hydrogel‐tissue chemistry for sample processing to secure biomolecules and support the spatial architecture of tissues. Barcode in situ targeted sequencing (BaristaSeq) utilizes Illumina sequencing‐by‐synthesis chemistry to read barcode sequences in situ with multiple rounds of imaging.^[^ ^34^ ^]^ The optimized protocol has shown improved signal‐to‐noise ratios, thereby enabling better detection during in situ synthesis of target‐specific RCA products (Figure 1d).

Since both FISH and ISS use pre‐designed probes to label target transcripts, only preset genes related to a certain experimental hypothesis are profiled, and this preset repertoire of target genes leads to biased detection of transcriptomes.^[^ ^35^, ^36^ ^]^ Although FISSEQ‐synthesized cDNA uses random hexamers and direct reading of cDNA sequences by SOLiD chemistry to avoid target selection bias and enable de novo discovery of expressed genes, it also shows low efficiency of gene detection owing to overwhelming occupancy of the fluorescence channels by signals originating from rRNAs (Figure 1e). Additionally, random hexamer priming results in a very poor yield after reverse transcription (0.2–1%), which inhibits the efficient detection of mRNAs. FISSEQ‐based methods also involve complex enzymatic reactions, a week of long imaging time, and challenging data processing owing to large raw data volumes. Therefore, despite mapping transcripts at the sub‐cellular resolution, imaging‐based technologies suffer from several technical limitations that hamper their clinical and biomedical applications for large tissues. In contrast, hybridization‐based ISS (HybISS)^[^ ^37^ ^]^ and STARmap‐based schemes allow the detection of signals by employing low magnification objectives (20×, numerical aperture (NA) 0.8 air for HybISS; 40×, NA 1.3 oil for STARmap), which enables the investigation of relatively large‐sized tissues.^[^ ^38^ ^]^ Directly targeting RNA with padlock probes to eliminate inefficient reverse transcription during cDNA synthesis can enhance the efficiency of ISS [barcoded oligonucleotides ligated on RNA amplified for multiplexed and parallel insitu analyses (BOLORAMIS)^[^ ^39^ ^]^ and hybridization‐based RNA ISS (HybrISS)^[^ ^40^ ^]^]. Moreover, commercialized options for imaging‐based spatial transcriptomics technologies will soon be available, including MERSCOPE^[^ ^41^ ^]^ (VizGen, Cambridge, MA, United States; MERFISH), Xenium^[^ ^42^ ^]^ (10X Genomics, Pleasanton, CA, United States; ISS and FISSEQ), products from Spatial Genomics (Pasadena, CA, United States; seqFISH), and GeoMX and CosMX from NanoString (Seattle, WA, United States).

### 2.2 Sequencing‐Based Methods

NGS platforms have shown unprecedented success in producing massive genomic sequencing data because of their superior sequencing capacity at a substantially reduced cost.^[^ ^43^ ^]^ The lack of spatial context in transcriptomic data, however, has led to the development of new techniques capable of encoding spatial information as nucleotide barcoding sequences. The spatial location of transcripts can be recovered using NGS by incorporating spatial barcodes to construct RNA sequencing libraries. NGS‐based spatial transcriptomics approaches have superior throughput compared to imaging‐based methodologies because the entire transcript information is detected and massively parallel‐processed. Additionally, NGS‐based approaches do not require a pre‐targeted list of genes because the retrieval of transcript spatial distribution is accompanied by cDNA synthesis in an unbiased and non‐targeted manner.^[^ ^35^ ^]^

Early approaches for including the spatial context in NGS performed microscopy‐guided selection and isolation of regions of interest (ROIs) for analysis by full‐read RNA sequencing. Laser capture microdissection (LCM) is a widely used strategy for the physical dissection of tissue ROIs by laser cutting.^[^ ^44^, ^45^ ^]^ LCM‐seq,^[^ ^44^ ^]^ and the more recent spatial‐histopathological examination‐linked epitranscriptomics converged to transcriptomics with sequencing (Select‐seq),^[^ ^45^ ^]^ combine LCM with Smart‐seq2 for polyA+ RNA sequencing of select cell populations that are identified for dissection by immunohistochemistry (IHC). Tomo‐seq uses a tomography‐inspired sectioning approach to resolve the transcriptome of anatomical ROIs.^[^ ^46^ ^]^ Through a combination of LCM‐seq and Tomo‐seq, Geo‐seq (geographical position sequencing) enables 3D transcriptome analysis of select ROIs and single cells.^[^ ^47^ ^]^ For an alternative to physical dissection, methods such as NICHE‐seq,^[^ ^48^ ^]^ ZipSeq,^[^ ^49^ ^]^ Light‐seq,^[^ ^50^ ^]^ and GeoMX Digital Spatial Profiling^[^ ^51^ ^]^ instead use optical methods to mark regions or cells of interest. The strength of these regioselective sequencing methods is easy implementation and sensitive sequencing with small sample sizes (<200 cells) where a clear spatial ROI is already defined by IHC staining. Additionally, LCM with SMART‐3Seq^[^ ^52^ ^]^ and GeoMX^[^ ^51^ ^]^ have demonstrated their applicability for formalin‐fixed paraffin‐embedded (FFPE) samples. Recent investigations employed GeoMX to study archived FFPE human cancer samples.^[^ ^53^, ^54^, ^55^ ^]^ Although there are notable advantages to LCM methods, they typically have limited spatial resolution and throughput compared to recent spatial barcoding NGS techniques.

**Figure 2:**
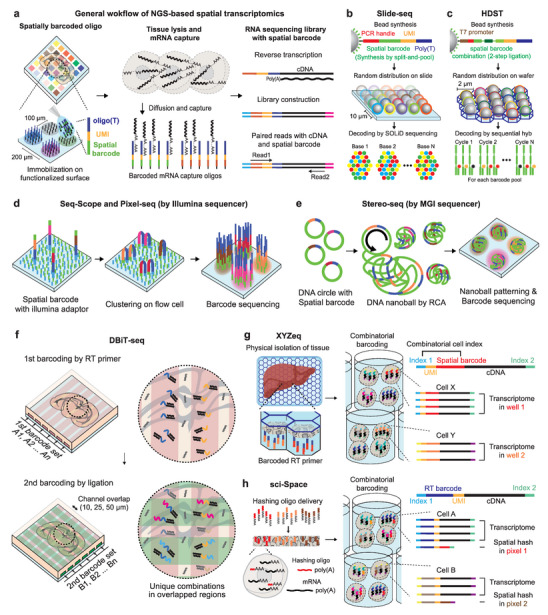
Next‐generation sequencing (NGS)‐based spatial transcriptomics methods. a) General workflow of the spatial transcriptomics (ST) method. The spatial barcode‐encoded oligos are immobilized on a functionalized surface to capture mRNA released from the mounted tissue or cells. Subsequent cDNA synthesis, followed by sequencing libraries yield transcript sequences and their spatial locations, simultaneously. b–e) Developments of methods for the spatial patterning of barcoded oligos with enhanced spatial resolution. Unlike imaging‐based ST, these methods require barcode decoding after patterning due to the random spatial distribution of spatial barcodes. b) Slide‐seq employs random spatial bead spreading and in situ sequencing decoding. c) HDST deposits beads with combinatorial barcodes on patterned wafers, followed by decoding with serial hybridization. d) Seq‐Scope and Pixel‐seq utilize Illumina clustering for oligo patterning and directly read sequences using Illumina sequencers. e) Stereo‐seq utilizes DNBSEQ chemistry to generate DNA nanoballs with spatial barcodes, which are patterned on a flow cell, and barcode calling is performed by the MGI sequencer. f) DBiT‐seq delivers barcoded RT primers and ligation oligos through orthogonal microfluidic channels. The predetermined spatial distribution of overlapping regions eliminates time‐consuming steps for random spatial barcode sequencing procedures. g) True single‐cell and single‐nucleus resolutions with regional spatial barcode printing in XYZeq. h) sci‐Space delivers hashing oligos with spatial barcodes into tissue followed by additional fixation to retain hashing oligos in the nuclei. After combinatorial barcoding for single‐nucleus RNA sequencing, hashing oligos are sequenced as a transcriptome.

In general, spatial transcriptomics describes techniques used for analyzing the spatial information of transcriptomes. According to a methodology reported earlier, spatial transcriptomics is a non‐generic term that has originated from the technique itself.^[^ ^56^ ^]^ This new method has been used to locate transcripts by transferring RNA molecules to a glass slide coated with poly‐T primers containing a unique molecular identifier (UMI) and a spatial barcode using ≈100 µm pixel size resolution (**Figure** **2**a). While capturing mRNAs with poly‐T on the slide surface, the newly synthesized cDNAs templated by these captured transcripts contain pre‐allocated spatial barcodes, which enable retrieval of the original transcript locations. Through library construction and NGS analysis, cDNAs and spatial barcodes can be sequenced to simultaneously identify and locate specific RNA transcripts in tissues. However, this strategy suffers from low RNA‐capturing efficiency and poor spatial resolution at a spot‐to‐spot distance of 200 µm and consequently lacks single‐cell resolution. Follow‐up techniques have been developed with an improved spatial resolution by reducing the pixel size of spatial barcodes (Figure 2b–e). Slide‐seq^[^ ^57^, ^58^ ^]^ has achieved a spatial resolution of 10 µm using a random distribution of barcode‐containing polystyrene beads on a slide (Figure 2b). High‐definition spatial transcriptomics^[^ ^59^ ^]^ (HDST) has subsequently demonstrated a spatial resolution of 2 µm using a silicon wafer (Figure 2c). However, the improved spatial resolution for Slide‐seq and HDST requires random distribution of beads containing spatial barcodes in space, and therefore, their exact distribution must be determined by time‐consuming imaging‐based in situ sequencing. Additionally, the detection efficiency is negatively affected by the mechanism of mRNA capture from even smaller sample volumes used with these methods.

To facilitate the fabrication of an mRNA‐capturing oligo array with encoded spatial barcodes, the Illumina NGS instrument can be employed to simplify the identification of spatial barcode distributions (Figure 2d). Seq‐Scope directly adopts the sequencing process utilized by Illumina NGS devices to generate spatial barcode arrays at a separation distance of ≈0.6 µm.^[^ ^60^ ^]^ Illumina sequencing libraries are generated containing both spatial barcodes and oligo‐dT, amplified by PCR, and dispersed on the flow cell through clustering of the sequencing device. Spatial barcodes are then identified through the actual sequencing steps. By treating tissue samples overlaid on the flow cell with digestive enzymes, the released mRNAs can be captured by the oligo‐dT domain anchored at the surface of the flow cell that also incorporates spatial barcodes. Similar Illumina chemistry employed in polony‐indexed library‐sequencing (Pixel‐seq)^[^ ^61^ ^]^ includes a modified polymer surface for clustering to minimize gaps between the barcoded pixels. This polymer‐based surface can generate continuous features by tightly distributed mRNA capture oligos. Subsequently, the spatial barcode is incorporated into the cDNAs by reverse transcription, and the cDNA sequence and spatial barcode are simultaneously analyzed by NGS. Spatial enhanced resolution omics sequencing (Stereo‐seq)^[^ ^62^ ^]^ also utilizes NGS devices to manufacture spatial barcode arrays using MGI's DNA nanoball sequencing (DNBseq) chemistry (Figure 2e). Stereo‐seq can localize the position of spatial barcode oligo arrays with a superior resolution at a diameter of ≈0.22 µm with a 0.5‐µm spacing in the DNBseq instrument. The UMI sequences are then ligated to mRNA‐capturing oligos for quantification. This ultrafine resolution increases the cost of sequencing an enormous number of pixel areas, thereby limiting large‐scale tissue investigation. In addition, at this ultrafine resolution, the lateral diffusion of mRNAs could lead to incorrect localization of transcripts by blurring their spatial locations.

Therefore, platforms that do not rely on delivering mRNA to a spatially barcoded oligo array anchored on the surface of chips or slides have been explored (Figure 2f–h). In deterministic barcoding in tissue for spatial omics sequencing (DBiT‐seq), oligos encoding spatial barcodes are directly delivered to fixed tissues using a microfluidic chip^[^ ^63^ ^]^ (Figure 2f). Oligos with predetermined spatial barcodes are loaded into each channel of the microfluidic chip and delivered to a certain grid position of the tissue. The same process is repeated along the axis of the fluidic channel that is perpendicular to the initial axis, and the resulting oligo mixtures at each grid position are ligated into cDNA in a combinatorial manner to produce spatial barcodes. The location where the two axes intersect is determined by examining the combinations of spatial barcodes by sequencing. Controlling the channel width of a microfluidic chip can also confer higher spatial resolution. Compared with Slide‐seq and HDST, each channel in DBiT‐seq contains a predetermined barcode sequence and does not require identifying the distribution of spatial barcodes using time‐consuming in situ sequencing. Furthermore, proteins can be labeled by treating fixed tissues with antibody‐oligo conjugates, which, with concomitant detection of nucleic acids, renders quantitative analysis of both proteins and mRNAs with their respective spatial distributions.

Labeling mRNAs with spatially barcoded oligos in compartmentalized microfluidic channels is still insufficient to reflect actual single‐cell‐level transcriptomes in space. Transcriptomes of relatively large pixels only provide mixed information on transcripts of multiple cells. By extension, inferred transcriptomes from multiple small pixels have inherent limitations in their correspondence to genuine single‐cell‐level data. The sci‐Space^[^ ^64^ ^]^ and XYZeq^[^ ^65^ ^]^ methods provide true single‐cell or single‐nucleus‐level spatial transcriptomes by dissociating cells or nuclei while preserving their spatial origins at a certain well‐ or pixel‐scale resolution (Figure 2g,h). After delivery of either hashing oligos containing spatial barcodes with poly(A) tail (sci‐Space, Figure 2h) or capturing mRNA by infiltrating tissues with spatially barcoded reverse transcription primers (XYZeq, Figure 2g), the single‐cell level transcriptome can be retrieved by inserting additional cellular barcodes via a non‐spatial version of combinatorial single‐cell or single‐nucleus indexing. However, these techniques suffer from RNA integrity issues because these protocols require chemical fixation to maintain cellular integrity during combinatorial indexing.

### 2.3 3D Spatial Investigation by Tissue Clearing and Expansion

Recent spatial transcriptomic technologies are restricted to 2D investigations of transcripts. Because NGS‐based methods use spatially‐barcoded oligo arrays to capture and label mRNAs to preserve their spatial origin, the tissue mRNA or spatially barcoded oligos must spread along the Z‐axis of tissues toward the surface in contact with the array. As a result, 3D spatial distribution is analyzed in a projected form on the 2D sample plate. In highly multiplexed FISH and in situ sequencing methods, the true 3D distribution of individual transcripts can be measured; however, imaging procedures involve time‐consuming microscopic measurements with very limited fields of view, such as the single optical plane that is analyzed in seqFISH+. Therefore, the collected data corresponds to a sequence of 2D samples, and local 3D spatial information is limited by the thickness of tissue sections. Although a 3D atlas can be constructed by serial sectioning, a systematic gap exists between the image data points because not all tissue sections can be subjected to imaging owing to the enormous volume of samples and the restricted throughput imposed by the imaging methods.

Tissue clearing techniques enable direct observation of 3D structures from transparent thick‐tissue blocks prepared by optical clearing and refractive index matching.^[^ ^66^ ^]^ In cleared tissues, lipids are removed to minimize light scattering inside tissues for optical clearing. Target proteins are labeled by immunostaining, however, owing to the harsh nature of tissue clearing protocols that involve organic solvents or highly concentrated aqueous solutions, tissue clearing methods may lead to damage or loss of RNA transcripts and are typically incompatible with RNA detection. For certain hydrogel‐based clearing methods, smFISH is compatible after lipid removal by detergents [CLARITY^[^ ^67^ ^]^ and passive clarity technique (PACT)^[^ ^68^ ^]^]. Additional RNA fixation helps to improve RNA retention during tissue clearing (EDC–CLARITY).^[^ ^69^ ^]^ MERFISH, an imaging‐based multiplexed FISH method, is also compatible with hydrogel‐embedded tissues and was previously employed to reduce autofluorescence signals from tissues. The tissue clearing method stabilization under harsh conditions via intramolecular epoxide linkages to prevent degradation (SHIELD)^[^ ^70^ ^]^ utilizes a polyepoxy crosslinker as a fixative and was originally developed to protect multimodal biomolecules and tissue architecture. RNA molecules are easily detected by FISH hybridization chain reaction (HCR) after tissue clearing by SHIELD; therefore, SHIELD could serve as a 3D spatial multi‐omic tissue processing and imaging platform.

Tissue expansion techniques allow super‐resolution imaging with diffraction‐limited light microscopy by physically expanding hydrogel‐embedded tissues. In expansion microscopy (ExM),^[^ ^71^ ^]^ swellable hydrogel‐embedded tissues expand and magnify their structure, which improves their effective resolution by increasing intermolecular distance. Following subsequent optimization of ExM using swellable hydrogels and a nucleic acid crosslinker (Label‐X), expansion FISH (ExFISH) has demonstrated multiplexed FISH in expanded biological samples.^[^ ^72^ ^]^ Further optimization of the ExFISH method led to expansion‐assisted iterative FISH (EASI‐FISH), which can quantitatively analyze relatively large tissue volumes (up to 300 µm thickness before expansion) to enable true single‐cell transcriptomics by investigating multiple layers of intact cells by imaging.^[^ ^73^ ^]^ Development of in situ sequencing using expansion methods yielded ExSeq^[^ ^74^ ^]^ for combining tissue expansion with in situ sequencing methods such as FISSEQ. Expansion sequencing (ExSeq) has further improved the resolution of FISSEQ by adding a physical expansion, resulting in better coverage of transcript detection in volume. Unified ExM (UniExM)^[^ ^75^ ^]^ employs glycidyl methacrylate (GMA) as a nucleic acid crosslinker, which dramatically reduces experimental costs. Using GMA improves nucleic acid retention in polyacrylamide‐based hydrogels and provides excellent results when applied in multiplexed FISH in situ sequencing with expansion. These 3D tissue investigation approaches may play significant roles in studying transcriptomes and multi‐omes of tissues and organoids as multi‐cell layer spatial information is necessary to understand the complex behavior of biological systems.

## 3 Challenges and Opportunities of Current Methods

### 3.1 Inherent Technical Difficulties in Imaging‐Based Methods

Highly multiplexed FISH and in situ sequencing methods are based on imaging using fluorescence microscopy to locate single transcripts at subcellular resolution. Although the spatial resolution varies among techniques, imaging‐based methods can provide sub‐micron resolution when employing a high‐NA objective during imaging. When additional information is obtained using probes [e.g., proteins labeled with antibodies and nuclei with nucleic acid‐labeling dyes such as 4',6‐diamidino‐2‐phenylindole (DAPI)], the spatial data can be projected onto the cell contours by cell segmentation. Although these imaging‐based methods provide high spatial resolution, this feature inevitably limits the size of tissue coverage. The tissue area captured within a single imaging cycle is limited by the field of view of the objective, and the imaging time depends on the signal‐to‐noise ratio and target resolution. In addition, detecting broad features from various transcriptional profiles requires multiple rounds of imaging followed by probe hybridization or enzymatic reactions for ISS. Acquiring multiple images includes time‐consuming steps, such as probe replacement between each image, and requires a substantial amount of time that limits the acquisition of information over a large area.

Optical crowding in raw images is another technical hurdle in imaging‐based methods. Considering that one cell is typically populated with thousands of transcripts, the point‐spread functions of the fluorescence signals emitted from these transcripts often overlap when simultaneously detecting multiple species in a single acquisition step. Highly multiplexed FISH methods can detect a large number of transcripts owing to their excellent hybridization efficiency; however, consequently, signal overlap makes the deconvolution of barcodes difficult. MERFISH and seqFISH+ can resolve optical crowding issues by reducing the number of transcripts detected in a single image providing increased space for barcode combination. However, this approach directly impacts imaging time by increasing the number of imaging rounds and time required to image large combinations of barcodes. In ISS‐based methods, optical crowding is less problematic because these methods exhibit low detection efficiency when converting transcripts to cDNAs in situ. FISSEQ detects ≈0.2–1% of transcripts owing to the low yield of in situ reverse transcription and cDNA synthesis. Other methods utilizing pad‐lock probes that hybridize to their target RNA species require enzymatic ligation that results in low detection compared to those of multiplexed FISH methods.

### 3.2 Inherent Resolution Issues in NGS‐Based Methods

NGS‐based methods generate massive amounts of raw data owing to the high throughput of NGS devices. Initial capturing of mRNAs using barcode oligos and subsequent cDNA synthesis does not require specialized equipment. An additional benefit is that NGS‐based methods use poly‐T oligos for unbiased capture of mRNAs, and therefore do not require predefined probe panels to label target RNA. This feature allows de novo discovery of RNAs with statistically significant differences in spatial distribution.

Owing to the limited spatial resolution, deciphering spatial barcodes to infer the spatial origin of transcripts cannot provide their cellular origin. The mRNA‐capturing oligos contain specific spatial barcode sequences that represent specific regions in 2D coordinates to distinguish the spatial origin of transcripts. A set of transcriptomes sharing the same spatial barcode constitutes transcriptome information for each tissue region, such as pixels in an image, and the arrangement of these pixels eventually represents the spatial distribution of the entire tissue transcriptome. However, each pixel specified by the same spatial barcode in the 2D coordinates does not provide the actual cellular boundaries required to reconstruct single‐cell‐level information. Therefore, different technologies require different information processing. If the size of a pixel is greater than that of a single cell, the transcript of one pixel would be a mixture of the transcripts of several cells and vice versa. Although sci‐space and XYZeq enable single‐nuclear distinction of the transcriptome by dissociating nuclei, the spatial resolution of nuclear location is still limited by pixel size.

### 3.3 Processing Speed for 3D Spatial Investigation with Tissue Clearing and Expansion

Tissue clearing and expansion techniques can enhance the effective resolution of 3D spatial transcriptomic investigations for future biomedical applications. Despite the valuable information that can be extracted through these methods, there are remaining points of optimization that continue to limit widespread implementation. To our best knowledge, the combinatorial barcoding scheme has not been applied for increasing multiplexity due to technical challenges in registration of fluorescence signals from large 3D raw images. As a result, the processing time required for repeated 3–4 color imaging and staining/destaining cycles with linearly increasing numbers of gene targets is lengthy for capturing large area samples. Additionally, the increased resolution and addition of volumetric analysis inherently result in immense data sets in biomedical applications. The current analysis platforms require considerable improvement before they are capable of handling data volumes in the order of a hundred terabytes to several petabytes. Following optimization of these aspects of tissue clearing and expansion techniques, large biomedical datasets with enhanced spatial resolution can be leveraged to uncover additional transcriptomic information from complex and crowded environments.

## 4 Challenges in Raw Data Processing

**Figure 3:**
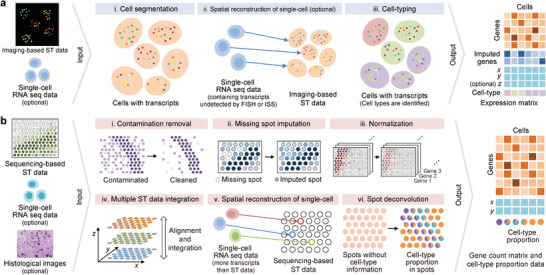
The workflows for preprocessing raw data in a) imaging‐based and b) sequencing‐based spatial transcriptomics methods. In the imaging‐based workflow, spatial transcriptomics data is predominantly analyzed independently, while single‐cell sequencing data is occasionally integrated. In contrast, sequencing‐based methods more commonly employ both single‐cell sequencing data and histological images simultaneously. The resulting outputs of the two workflows differ in their celltype format. For sequencing‐based methods, the spot size is usually larger than a single cell, so the cell type of each spot is described proportionally. In contrast, imaging‐based methods provide a cell‐level gene count matrix, which directly labels the cell type on each cell. These outputs are then employed for downstream analysis.

After successfully implementing spatial transcriptome experiments, interpreting large amounts of raw gene expression matrices is challenging. Each method for spatially resolved transcriptomics generates data of various scales, resolutions, and modalities, according to their working mechanisms, to capture and identify transcripts. Therefore, analytical pipelines that process raw data should consider these differences for successful interpretation. Additionally, limited information on current spatial transcriptome data is sometimes analyzed together with the existing information. In this section, we introduce useful analysis pipelines and algorithms for handling data from spatial transcriptomics (**Figure** **3**). Since imaging‐based and sequencing‐based methods generate raw data in different classes, the workflows are depicted in two categories.

### 4.1 Dealing with Error‐Prone Raw Data

Regardless of their working principles, all imaging‐based methods for spatial transcriptomics require three crucial steps in raw data processings: 1) registration of barcoded fluorescence signals from raw images, 2) barcode calling or processing to assign target RNA reads, and 3) efficient cell segmentation to assign barcoded dots to each cell. Prior to further processing, raw images are stitched and maximum‐projected along the z‐axis to generate 2D *xy* plane images. These images are typically subjected to various filters, such as Laplacian of Gaussian filters to remove noise and thresholding for dot detection. Initially, coarse‐grained registration among color channels and imaging rounds is applied with reference to specified registration markers (e.g., fluorescence beads or blood vessel staining). One significant challenge in assigning barcodes is to determine the acceptable error range in terms of pixel distances for successful barcode calling with minimal error. When the number of barcoding genes is small, less accurately registered fluorescence signals may be tolerated without substantially affecting the success rate in barcode calling. Some methods such as MERFISH have error correction schemes to reduce the effect of incorrect barcode assignment. However, transcriptome‐level barcoding requires raw images obtained through super‐resolution imaging modalities to further resolve multiple transcripts located within diffraction‐limited spots. With this level of complexity, barcode calling rates can be significantly reduced compared to those of experiments with dozens and hundreds of target genes, and the resulting analysis scheme inherently yields decreased RNA detection efficiency. Imaging‐based methods also involve image processing and error correction steps for chromatic correction between fluorescent channels and image registration between multiple rounds. For example, initial MERFISH papers employed only the 647 channel to avoid chromatic aberration.

It is important to note that all of the correction steps mentioned earlier are typically performed with specific ad hoc parameters optimized for a particular spatial transcriptomics method and setup, which makes the raw image data processing pipeline difficult to generalize. Therefore, it may be more practical for researchers to begin their imaging‐based spatial transcriptomic analysis with pre‐processed data generated by commercial or pre‐optimized protocols. The following sections will focus more on reviewing data‐processing techniques for NGS‐based methods.

Due to technical limitations of currently available NGS‐based spatial transcriptomic methodologies, raw data may contain noise from various sources and often suffer from signal loss. As a result, a low signal‐to‐noise ratio (SNR) can compromise the accuracy of data analysis. To address this issue, preprocessing methods for noise reduction have been explored.

The datasets generated by NGS‐based methods, such as 10X Visium, contain spot swapping, where a certain spot may contain transcripts from nearby spots. These unwanted contaminants can be removed using a probabilistic model called SpotClean^[^ ^76^ ^]^ [Figure 3b(i)]. Due to the low transcript‐capturing efficiency of NGS‐based spatial transcriptomic methodologies, raw data suffer from loss of gene expression information. Thus, data imputation methods that are specifically tailored to the characteristics of the spatial data can enable the combination of spatial spots with corresponding pathological images^[^ ^77^ ^]^ and provide consolidated analysis by spatial transcriptomics and pure single‐cell sequencing data^[^ ^78^, ^79^ ^]^ [Figure 3b(ii)].

Normalization of spatial transcriptome data is crucial for comparing gene expression between spots or genes [Figure 3b(iii)] and is performed using conventional methods (e.g., regularized negative binomial regression).^[^ ^80^ ^]^ Recently, using deep‐learning models, morphological features have been extracted using spatial gene expression patterns in combination with hematoxylin and eosin (H&E)‐stained images. The extracted features can be compared among spatial data spots to complete the normalization of spatial data, which is called Spatial Morphological gene Expression Normalization (SME Normalization).^[^ ^81^ ^]^

### 4.2 Integration of Spatial and Single‐Cell Transcriptome Data

#### 4.2.1 Predicting the Spatial Distribution of Transcripts

Single‐cell and single‐nucleus sequencing databases have grown rapidly in terms of higher throughput and data quality. When consolidating these rich databases, it is important to predict the spatial distribution of transcripts by combining relatively crude quality spatial data [Figure 3b(iv)]. Spatially reconstructed transcriptomic data already provides valuable information, but single‐cell sequencing data can be used to address some of the limitations of spatial transcriptomics methods. One drawback of FISH or ISS data is that it only includes preselected genes and excludes others. These excluded genes can be imputed using spatially reconstructed single‐cell sequencing data because scRNA‐seq can profile all types of transcripts using poly‐T or random primers [Figure 3a(ii)]. In the case of sequencing‐based spatial transcriptomics data, it often suffers from a low number of captured genes. Integrating single‐cell sequencing data can improve this, as single‐cell sequencing data typically contain a sufficient number of genes [Figure 3b(v)].

Seurat^[^ ^82^ ^]^ allows seamless integration between single‐cell sequencing data and multiplexed FISH data by comparing the expression of landmark genes in single‐cell sequencing data with their spatial distribution in multiplexed FISH data. The expression data obtained from this process is represented by a bimodal mixture model. Spatial backmapping^[^ ^83^ ^]^ adopts a similar approach, but comparisons are based on specificity‐weighted mRNA profiles, which indicate the expression of each gene in a specific cell relative to that of all other cells.

Unlike these methods focusing on the spatial reconstruction of single‐cell sequencing data, spatial gene enhancement (SpaGE)^[^ ^78^ ^]^ imputes missing genes in multiplexed FISH data using single‐cell data via principal component analysis (PCA)‐based domain adaptation and k‐nearest‐neighbor regression. Gene imputation with Variational Inference (gimVI)^[^ ^79^ ^]^ method also performs imputation using a deep generative model.

Recently, data for the integration of spatial contexts is more diversified, and deep learning is widely employed. Seurat v3^[^ ^84^ ^]^ integrates single‐cell and spatial data, as well as chromatin accessibility and immunophenotyping data. Integrative analysis of multi‐omics at single‐cell resolution (GLUER)^[^ ^85^ ^]^ integrates single‐cell sequencing data with transcript and protein spatial data captured by highly multiplexed methods such as co‐detection by indexing (CODEX)^[^ ^86^ ^]^ and MERFISH.^[^ ^22^ ^]^ Both DEEPsc^[^ ^87^ ^]^ and Tangram^[^ ^88^ ^]^ employ single‐cell sequencing data for spatial reconstruction and can consolidate with spatial data obtained by spatial transcriptomics methodologies without limitations; therefore, both multiplexed FISH and NGS‐based data can be used as inputs. GLUER, DEEPsc, and Tangram have recently started using deep neural network models for improved prediction and data integration.

#### 4.2.2 Cell‐Type Inference and Spot Deconvolution

Performing cell‐type‐based analysis is challenging when the spatial resolution of a method is lower than the size of a single cell because in this situation multiple cells can contribute to the transcripts extracted from each spot. Therefore, it is essential to estimate the proportion of cell types in each spot through deconvolution using nonspatial single‐cell sequencing data [Figure 3b(vi)]. Because the spot size of recent NGS‐based spatial techniques is larger than the size of a cell, deconvolution methods for spatial transcriptomic data have been actively proposed since 2020. On the other hand, Slide‐seq, HDST, Seq‐Scope, and Pixel‐seq have finer resolutions; therefore, the spot size is comparable to a cell or even smaller. For deconvolution, statistical models or matrix decomposition can be employed,^[^ ^89^, ^90^, ^91^, ^92^, ^93^, ^94^ ^]^ but treating deconvolution by substituting it as a domain adaptation task is also plausible.^[^ ^95^, ^96^ ^]^ These methods initially define cell type as discrete or solid. Based on this hypothesis, they carry out cell‐type inference. However, tissues with cancer and inflammation may contain transcriptome variations in each cell type. Deconvolution of spatial transcriptomics profiles using variational inference (DestVI) conducts cell‐type inferences based on a continuous cell‐type model.^[^ ^90^ ^]^ While most spot deconvolution methods estimate the proportion of different cell types within a given spot, the results do not provide single‐cell level resolution. In contrast, cellular spatial positioning analysis via constrained expression alignment (CytoSPACE) employs single‐cell sequencing data to assign each cell to a specific spot in spatial transcriptomic data, thereby achieving single‐cell level resolution.^[^ ^97^ ^]^

Detailed benchmarking of the performance of spatial and single‐cell transcriptome integration has recently been published.^[^ ^98^ ^]^ Notably, performance benchmarking may be heavily affected by data volume, particularly in deep learning models. Additionally, the spatial resolution of each method significantly affects the performance of the cell‐type inference. For example, robust cell type decomposition (RCTD)^[^ ^91^ ^]^ and stereoscope^[^ ^92^ ^]^ use a direct‐count model for their inference; therefore, better performance is expected on high‐resolution spatial data.^[^ ^99^ ^]^

When performing cell type detection, certain cell types may not be identified if the corresponding cells or transcripts are not captured in the single‐cell sequencing data or spatial transcriptomic data. This can result in misinterpretation of the overall results, especially if the cell type of interest is rare. Therefore, to improve the reliability of a spatial transcriptomics analysis, a power analysis framework has been proposed.^[^ ^100^ ^]^

### 4.3 Alignment and Integration of Multiple Spatial Data

Currently, NGS‐based methods may exhibit poor transcript‐capturing efficiency. The computational strategy used for improvement is to collect multiple adjacent 2D spatial transcriptome datasets from tissues and then perform alignment and integration from multiple 2D data points [Figure 3b(iv)]. Here, alignment means finding pairwise spots (i.e., overlapping zones) between 2D data points, and the integration constructs single‐domain 2D data by consolidating multiple 2D data. As a result, these integrated spatial transcript data contain more gene expression information than that of the source data. In STUtility, histological images obtained from the same tissue used for spatial transcriptome data acquisition are employed for 2D data alignment.^[^ ^101^ ^]^ The probabilistic alignment of ST experiments (PASTE) method is effective without employing histological images.^[^ ^102^ ^]^ Instead, PASTE finds pairwise alignment between several 2D datasets based on probable transcriptomic and spatial resemblance.

### 4.4 Cell Segmentation and Cell Typing of Imaging‐Based Methods

Analyzing data produced by imaging‐based methods requires a cell segmentation task for classifying transcripts to each assigned cell [Figure 3a(i)]. Approaches to cell segmentation tasks for imaging‐based methods can be classified into three major categories: 1) manual, 2) supervised image segmentation, and 3) unsupervised approaches. It is worthwhile to note that conventional image processing approaches without machine‐learning models show inferior accuracy compared to supervised image segmentation techniques and are rarely studied nowadays. With this consideration, conventional approaches have not been discussed further in this paper.

Manual and supervised image segmentation approaches first fluorescently stain cell bodies and/or nuclei, and then recognize cell and/or nuclei boundaries by manual or supervised algorithms. Manual approaches require huge amounts of labor and the results are often unsatisfactory. Commonly, cell boundaries are not clearly distinguishable from multiplexed FISH or ISS images. Supervised image segmentation requires a manually annotated dataset for training machine learning models,^[^ ^103^, ^104^, ^105^, ^106^ ^]^ often resulting in inaccurate segmentation for other datasets due to the low performance of the generalized models.

Unsupervised approaches utilize transcript distribution to cluster transcripts for assignment to each cell. These approaches could be adequate alternatives or complements to previously discussed cell segmentation approaches because they are annotation‐free and often show better performance on cell‐level transcripts clustering as transcript distribution can be a better indicator of segmentation than visually recognizable cell boundaries in messy fluorescence images.^[^ ^107^ ^]^ Also, supervised image processing and unsupervised transcript clustering approaches can be used together to complement each other.^[^ ^108^, ^109^ ^]^

By analyzing the imaging‐based spatial transcriptomics data, cell types can be also identified [Figure 3a(iii)]. This can be conducted with data obtained purely from imaging‐based methods without aids from sequencing data. In these approaches, cell types are defined within the cell segmentation results.^[^ ^107^ ^]^ Hence, inaccurate cell segmentation results in poor cell type identification. This issue can be resolved by employing a cell segmentation‐free method, which uses spots as a unit of cell‐type inferences.^[^ ^110^ ^]^ Additionally, single‐cell sequencing data can be combined to identify cell types from FISH or ISS data more precisely.^[^ ^36^ ^]^

### 4.5 Future Works for Analyzing Spatial Transcriptomics Data

Spatial data processing studies focus on single‐cell or supracellular‐scale data, probably obtained using NGS‐based methods. However, imaging‐based methods can obtain subcellular‐level details; therefore, subcellular‐level processing of the spatial distribution of transcripts will be highly important. Currently, the database may be too limited to employ deep learning approaches, as found in earlier studies by Seurat and gimVI for <2000 cells. Therefore, the performance of analysis is not guaranteed, and more experimental databases are expected to be available to develop algorithms.

## 5 Application Note in Neuroscience

**Figure 4:**
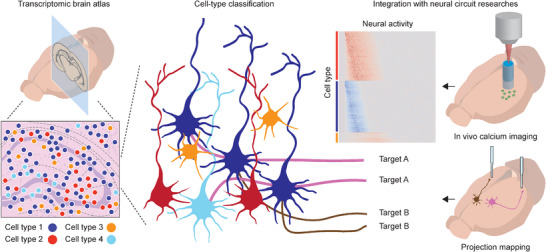
Applications of spatial transcriptomics in neuroscience. The transcriptomic atlas is established by cell‐type identification with the spatial context of precisely compartmentalized brain structure. Transcriptome‐based neuronal cell types can be further dissected by integrative studies employing tools for functionality and connectivity investigations. Accurate neural classification will lead to circuit‐level studies to investigate individual circuits to elucidate the comprehensive function of the entire brain.

The nervous system of higher organisms has a complex structure. By orchestrating organism‐level responses to internal or external stimuli, the nervous system governs complicated communications across the peripheral and central nervous systems. Because the function of the nervous system is primarily determined by the connections between functional neurons, a comprehensive understanding of this system requires elucidation of the wiring between different types of neurons by dissecting neuronal communication. Spatial transcriptomics can provide important information regarding the molecular states of cells, which can be integrated with various physiological features by precise assignment of individual cells (**Figure** **4**).

### 5.1 Brain Transcriptomic Atlas

The brain is the most segmented and annotated organ according to its spatial architecture. The brain is precisely divided into regions or areas associated with specialized functions and connectivity. However, as brain regions become more fragmented, the accuracy of defining their entities becomes controversial.^[^ ^111^ ^]^ Spatial transcriptomic analysis on a series of coronal sections across the whole brain provides new borders for region annotation defined by molecular characteristics. The hierarchical clustering of spatial transcriptomic data points is highly correlated with conventional neuroanatomical annotation and provides novel spatial borders to segment molecular subregion candidates.^[^ ^112^ ^]^ To establish a single‐cell spatial atlas with cell‐type annotation, Zhang et al.^[^ ^113^ ^]^ profiled ≈300 000 cells in the primary motor cortex of mouse brain using MERFISH with a selected gene panel derived from the results from previous single‐cell RNA sequencing (scRNA‐seq) and single‐nucleus RNA sequencing (snRNA‐seq). Manno et al.^[^ ^38^ ^]^ demonstrated the potential to create a spatial atlas of developing mouse embryos by integrating scRNA‐seq data with in situ sequencing of transcripts using HybISS applying a deep‐learning‐based method.^[^ ^88^ ^]^ STARmap PLUS established a spatial molecular atlas with single‐cell resolution by successful segmentation from transcript annotation,^[^ ^114^ ^]^ from which the imputed gene expression pattern of a brain section was found to be comparable to the in situ hybridization (ISH) database of Allen Mouse Brain Atlas.^[^ ^115^ ^]^ STARmap PLUS also enables the study of engineered recombinant adeno‐associated virus (rAAV) tropism across whole mouse brain regions by capturing transcripts packaged in AAVs. Given that the mouse brain atlas is not merely an architectural map of the brain but also contains various functional annotations, integrating functionality with the molecular atlas is noteworthy for further improvements. The Brain Initiative Cell Census Network has initiated the integration of multiple transcriptome data generated by different methods, samples, and laboratories to construct a multimodal atlas of the primary motor cortex while tracking the laminar distribution of diverse neuronal types using MERFISH.^[^ ^116^ ^]^ Allen Institute for Brain Science recently announced the creation of an extensive spatial transcriptomic atlas covering the entire adult mouse brain. This achievement was made possible by integrating data from scRNA‐seq analysis of 7 million cells and MERFISH analysis of 4 million cells, resulting in a total of nearly 11 million cells analyzed.^[^ ^41^ ^]^ The resulting atlas is an unprecedented achievement that sheds light on the spatial organization of individual cell types and validates the role of transcription factors in determining cell type through an integrated hierarchical classification of spatial transcriptomics. Building a comprehensive reference atlas is a critical endeavor that will facilitate the generation and validation of new hypotheses as spatial transcriptomics continues to uncover novel transcriptional relationships with functions and architectures.

### 5.2 Neural Classification

The classification of neuronal types can facilitate investigating the comprehensive relationship between the structure and function of individual cells. Various criteria are applied to dissect neurons for proper classification based on their morphology, physiology, and molecular features.^[^ ^117^ ^]^scRNA‐seq has been widely employed in the molecular profiling of neurons to identify specific molecular characteristics that correlate with their physiology and function.^[^ ^118^, ^119^, ^120^, ^121^, ^122^, ^123^ ^]^ Spatially resolved transcriptomics can provide new insights for criteria to tabulate ambiguous cell types into different categories according to their origins.

Connectivity, another criterion for neuronal classification that was merely accessible previously owing to the lack of efficient methodologies,^[^ ^117^ ^]^ can be analyzed through a spatially resolved transcriptomic investigation. In barcode analysis by sequencing (BARseq),^[^ ^124^ ^]^ the anterograde tracing virus packaged with random RNA barcodes was injected into the cortical area, and the barcodes were sequenced by in situ sequencing using BaristaSeq.^[^ ^34^ ^]^ The single‐cell projection pattern was identified by matching RNA barcodes and barcode reads using bulk‐RNA sequencing in multiple projection areas. The gene expression pattern from smFISH was integrated to identify the cell types of projection‐mapped neurons. BARseq2,^[^ ^125^ ^]^ an improved version of BARseq, allows multiplexed gene detection with padlock probes and in situ sequencing, followed by mapping of projection patterns to broad molecular profiles with single‐cell resolution.

The landscape of isoform expression is modulated by alternative splicing as post‐transcriptional regulation of gene expression, thereby contributing to the phenotype of individual cells. Therefore, different isoforms can provide various criteria for subdividing cell types identified by gene expression profiling. The conjunction of high‐throughput single‐cell gene expression profiling by 3′‐end sequencing and full‐length RNA‐seq could subdivide clusters that were previously identified by RNA profiling based on their isoform expression patterns. The gradient distribution of isoform landscape in the primary motor cortex of the mouse brain was observed using MERFISH, with marker genes correlated with isoform expression.^[^ ^126^ ^]^ Joglekar et al.^[^ ^127^ ^]^ directly observed the spatial distribution of isoforms by long‐read sequencing of RNAs captured by 10X Genomics Visium.

### 5.3 Studying Neural Circuits

Neural circuits are composed of substantially intermingled neuronal connections across the entire nervous system. Dissecting a specific circuit to study the functions of individual neurons is a fundamental approach to comprehending the entire system. The functional connectivity of neural circuits is being explored with developed genetic tools for dissecting neural circuits according to their phenotypic properties.^[^ ^128^, ^129^ ^]^ For instance, in vivo calcium imaging enables functional observation of neurons even within freely moving animals.^[^ ^130^, ^131^ ^]^ Spatially resolved neuronal functionality and transcriptomics integrate different modalities of neuronal phenotypes to further dissect neuronal identities.^[^ ^132^, ^133^ ^]^ Post‐hoc molecular profiling of the recorded cells can be carried out using IHCof *ex vivo* brain sections of the same animals, enabling the detection of marker genes to identify neuronal subtypes.^[^ ^134^, ^135^, ^136^ ^]^ In this regard, rather than simply detecting a few marker proteins by IHC, multiplexed FISH can further enhance the performance of molecular profiling by analyzing the higher number of markers to classify neurons of interest according to their expression patterns.^[^ ^137^, ^138^, ^139^ ^]^ Bugeon et al.^[^ ^140^ ^]^ developed combinatorial padlock‐probe‐amplified FISH (coppaFISH) for multiplexed FISH to detect 72 genes in the primary visual cortex of mouse brain for posthoc transcriptomic profiling after in vivo calcium imaging during visual stimulation. This increased number of detected transcripts enabled the precise classification of individual cells based on the previous scRNA‐seq transcriptome. The dependency of neuronal responses to visual stimuli according to the cortical state was related to the transcriptional profile of cells.

Moffit et al.^[^ ^25^ ^]^ characterized the molecular profiles of neurons activated by social behavior using MERFISH. Representative marker genes for MERFISH analysis were selected after scRNA‐seq of the preoptic area, which is a subregion of the hypothalamus, and MERFISH analyzed the spatial organization of each cell type in this area. By integrating neuronal activities derived from immediate early gene expression with cellular transcriptome, the transcriptomic profile of behavior‐related neurons and their locations were identified. The analysis subdivided predefined cell types into precise classes and provided anatomical information for socially relevant neural circuits.

### 5.4 Pathology

Pathology of the neural system is extremely complex owing to cellular heterogeneity and complex interactions among resident cells. Spatially resolved transcriptomics offers new perspectives on how these interactions influence the study of pathological mechanisms. In mechanistic studies with animal models, spatial transcriptomics can validate specific hypotheses of causality of diseases with spatial context^[^ ^141^, ^142^, ^143^ ^]^ or directly characterize a new concept to find disease‐related features from spatial correlation.^[^ ^144^, ^145^ ^]^ Chen et al. is a representative study demonstrating how to generate and validate new hypotheses concerning correlations between genetic signatures and pathological structures related to Alzheimer's disease. Novel genetic signatures for Alzheimer's disease were suggested by the identification of gene network alterations in the periphery of *β*‐amyloid plaques using low‐resolution spatial transcriptomics and characterization of cellular identity for cells possessing altered gene expression using ISS.^[^ ^146^ ^]^

Studying pathology in the human brain requires precise targeting, as its physical scale generally exceeds the currently available biochemical tools used for small animals, and these procedures require pathologists who can distinguish between physiologically normal and diseased regions within a specimen. This encourages the application of spatial transcriptomics to clinical specimens to characterize the biological features that can be discriminated from a diseased partition of tissues compared to adjacent healthy tissues. Candidate genes associated with hereditary amyotrophic lateral sclerosis can be identified by detecting cerebellar granule cell layer‐specific transcripts in H&E‐stained sections of post‐mortem tissue specimens.^[^ ^147^ ^]^ Kaufmann et al.^[^ ^148^ ^]^ identified the T‐cell subtype crossing the blood‐brain barrier to colonize the brain and cause autoimmune multiple sclerosis and observed the residence of T‐cells inside the brain during disease progression using spatial transcriptomics. The same group identified putative drug targets for progressive multiple sclerosis with interactome analysis based on transcriptome distance derived from spatial transcriptomics.^[^ ^149^ ^]^

### 5.5 Future Perspectives in Neuroscience Application

Spatial transcriptomics has impacted various fields of neuroscience by providing additional spatial dimensions to other existing research modalities. As the application of spatial transcriptomic technology in neuroscience matures, spatially resolved transcriptome data will be further consolidated with separate analytical modalities. Applying spatial transcriptomics to the investigation of the brain during different developmental stages has enabled spatiotemporal tracking of transcriptomic changes in specific developmental structures, allowing us to impute the intermediate cellular and molecular profiles during neurodevelopment processes.^[^ ^150^ ^]^ By integrating rich scRNA‐seq data with limited gene profiles generated by seqFISH, entire gene expression patterns were successfully inferred, and this provided additional gene profiles related to the organization of developmental processes, specifically those associated with the formation of the midbrain‐hindbrain boundary.^[^ ^151^ ^]^ By integration of data from spatial transcriptomics and imaging of dynamics of chromatin structure, it has been suggested that chromatin condensation is a predictive criterion for Alzheimer's disease progression.^[^ ^152^ ^]^ As we found in these examples, spatial transcriptomics has the potential to provide high‐dimensional molecular profiling to conventional neurodevelopmental and neuropathological investigations. Since neuroscience is a multidisciplinary field with various tools available for research, the addition of spatial information using spatial transcriptomics is expected to dramatically expand the boundaries of existing knowledge.

Spatial transcriptomics is still a new approach in neuroscience research, particularly for clinical studies. By enabling high‐throughput molecular profiling with spatial contexts, it will offer a unique opportunity to comprehend complex biological systems composed of intricate cell‐to‐cell interactions. Additionally, spatial transcriptomics holds promise as a valuable screening tool to enhance disease diagnosis accuracy, which will be further discussed in the following section.

## 6 Application Note in Cancer Studies

**Figure 5:**
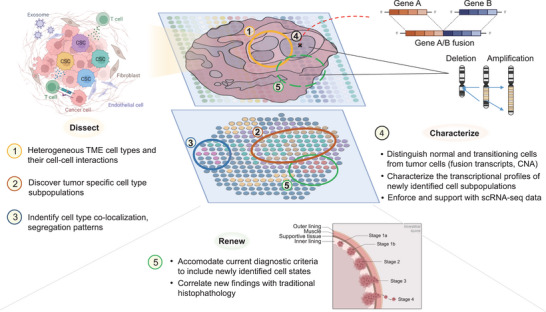
Applications of spatial transcriptomics in cancer research. Spatially resolved transcriptomics can be used to define the cell type compositions and discover new cell–cell interactions of specific tumor ecosystems, profile and characterize these ecosystems by utilization and integration in multi‐modal/omics analyses, and help fuel the joint renewal of current histopathological standards to accommodate these new findings. (Figure created with icons and redesigned templates provided by Biorender.com).

Heterogeneity increases as cancer transitions from a benign to malignant state. The increasing heterogeneity hinders the prevention and treatment of cancer. Cellular components of the tumor immune microenvironment (TIME) and communication between cells in the TIME are associated with cancer prognosis and response to therapies.^[^ ^153^ ^]^ Spatial transcriptomics can help clinicians and scientists discover new cell types and co‐localization patterns, characterize the TIME, and monitor tumor response to therapy (**Figure** **5**).

By linking spatial transcriptomes with scRNA‐seq data, new cell types can be identified, and cellular interactions can be inferred from cell‐type co‐localization patterns. In a study of human cutaneous squamous cell carcinoma (cSCC), researchers identified a specific cell type, tumor‐specific keratinocytes (TSKs), crowding the leading edge of the tumor and confirmed a surrounding fibrovascular niche consisting of cancer‐associated fibroblasts (CAFs) and endothelial cells.^[^ ^154^ ^]^ This confirmation of spatial transcriptomics colocalization was used to support inferences on cell interaction drawn from scRNA‐seq, and subsequently, TSKs were found to be a pillar of intercellular communication. Some immune cells were also found to possibly hinder effector lymphocytes from accessing the tumor. These discoveries showcase the potential of spatial transcriptomics for discovering targets for therapeutic intervention by immune control. Overall, the cell subpopulations constituting the cSCC tumor and stroma, and interaction among their spatial niche could be characterized. Another study reinforced previous knowledge that epithelial‐to‐mesenchymal transition (EMT) and proliferation are inversely correlated—for example, an exit from the EMT is required to enter proliferation, by reporting the cellular relationship of cells of the two states. Cell states related to the respective processes were segregated into their own distinct zones, the regions being mutually exclusive.^[^ ^155^ ^]^ In the case of pancreatic neoplasms, spatial transcriptomics profiling directly confirmed the initial annotations of pancreatic intraepithelial neoplasia constructed with scRNA‐seq data.^[^ ^156^ ^]^ Furthermore, receptor‐ligand analyses suggested the interaction of the T cell immunoreceptor with Ig and ITIM domains (TIGIT) receptor of lymphocytes with Nectinligands, TIGIT being highly expressed in tumor cells. The spatial results help to weigh in on the speculation that tumor cells could exploit TIGIT‐Nectininteraction to evade the immune response.

Spatial transcriptomics is emerging and gradually gaining recognition as an essential tool for sifting through the tumor microenvironment. A spatial transcriptome constructed from 21 tissues progressing from non‐tumor to ecotone, to tumor regions of primary lung cancer revealed that the tumor capsule was involved in transcriptome complexity, immune cell infiltrations, and continuity of intratumor spatial clusters; intratumor architecture was supported by bidirectional ligand‐receptor signaling at the cluster perimeters; and PROM1^+^, CD47^+^ cancer stem cell niches contributed to the remodeling of the tumor microenvironment and metastasis.^[^ ^157^ ^]^ Hunter et al. described an “interface” cell state located at the ecotone of tumor and normal tissues, including the possibility that this new cell state induces tumor invasion into surrounding tissues.^[^ ^158^ ^]^ Although the sequencing resolution and depth of spatial transcriptomic data may not be comparable to those of scRNA‐seq data, sufficient spatial data are preserved for linking and deconvoluting the spatial transcriptomic data with scRNA‐seq data. Analysis of spatial transcriptome data can indicate specific transcriptomic signals originating from the location of cellular aggregation and enables additional insights into cell types and interactions that may assist TIME formation. This can help determine whether specific cell–cell interactions are enhanced in segregated niches or separately organized structures.^[^ ^155^, ^159^, ^160^ ^]^

Spatial transcriptomic data along with scRNA‐seq have become routine procedures in exploring and characterizing tissues, cell types, and diseases. Consequently, spatially resolved multi‐omics studies will be increased in cancer biology. A combination of scRNA‐seq, spatial transcriptomics, genomics, and metabolomics was used to reveal spatially segregated transcription patterns, which revealed distinct genomic alterations and identified hypoxia as a driver for genomic instability.^[^ ^160^ ^]^

The inference of copy number alterations (CNAs) using spatial transcriptomics is consistent with bulk whole‐exome sequencing (WES) data, thereby reinforcing its practicality.^[^ ^157^ ^]^ Distinct clonal patterns of spatial CNAs are found near and within tumors and could be used to distinguish tumors from normal and transitional cell states. These clonal CNA patterns located in cancer drivers are not associated with immediately visible morphological transformation; therefore, they are strongly proposed as a measure for early diagnosis of cancer.^[^ ^161^ ^]^

Novel transcripts, such as fusion transcripts and alternative splicing variants, are frequently observed in various cancers. Spatial transcriptomics fusion (STfusion)^[^ ^162^ ^]^ can infer the existence of fusion transcripts from spatial transcriptomic data. By applying this method to prostate cancer, cis‐SAGe *SLC45A3‐ELK4* has been detected mostly in physiologically altered, inflamed, or neoplastic areas. Long‐read sequencing that enables the profiling of full‐length transcripts has been applied to spatial transcriptomics and scRNA‐seq.^[^ ^163^ ^]^ Full‐length spatial transcriptomics can be used to explore alternative splicing events during tumorigenesis.

Recently, base‐specific in situ sequencing (BaSISS) of mutation branches that were obtained from whole genome sequencing was utilized to simultaneously track the cancer‐specific somatic mutations arising in breast cancer.^[^ ^164^ ^]^ Spatial transcriptomics was integrated to generate maps that quantitate these genetically distinct subclones. Genetically similar subclones were found to exhibit similar co‐localization patterns and histological features regardless of their spatial vicinity, in the same way, distinct subclones localized with different groups of immune cells, possibly mediating clone‐specific immune interactions.

The discovery of new factors influencing tumorigenesis and characterizing malignancy using spatial transcriptomics will bring new diagnosis criteria and standards for cancer research. As the niches of TIME, cell‐type interactions, and cancer expression regions become unveiled, they deviate from traditionally annotated regions of cancer.^[^ ^165^ ^]^ The future of automating the process of histopathology‐based diagnosis is near, especially with the advent of machine learning technology and artificial intelligence.^[^ ^166^, ^167^, ^168^, ^169^, ^170^ ^]^ These technology‐based methods are trained on pathologist‐annotated histology slides and spatial transcriptomics data, then are able to distinguish healthy and diseased areas of the tissue, infer gene expression levels to spatially characterize tumor heterogeneity, and detect expression‐supported morphological patterns that are indistinguishable to the human eye. The application of a deep learning method trained on whole slide images to breast and lung cancer slides deduced a statistically significant link between high tumor heterogeneity and poor survival.^[^ ^167^ ^]^ A neural network‐based method trained to relate histological morphology to the underlying gene expressions was not only able to generally match the manual assessments by pathologists but also provided more detailed interpretation, taking the actual expression patterns into account.^[^ ^168^ ^]^ These techniques can detect and classify specific tumor subregions, streamlining the laborious task of manual annotation. Additionally, they can be quickly trained on large datasets and minimize potential errors and variations that may arise from human annotation. These advantages will aid in making better decisions regarding the specific treatment type for matching specific disease stages or subtypes. With the support of future research, existing histopathological diagnosis criteria may need to undergo minor to major updates to accommodate the new findings presented by spatial transcriptomics.

The use of spatial transcriptomics holds promise in cancer detection and subtype classification, however at its current state is in need of more case studies, data, and process standardization before its use as a clinical classifier.^[^ ^166^, ^171^ ^]^ With more spatial cancer data acquired through movements such as the Human Tumor Atlas Network launched by the US National Cancer Institute in 2018 to construct cellular, morphological, and molecular features of progressing cancer, and with higher resolution, researchers will be more equipped to identify the key genes and regulators in the processes of mutation acquisition, factors differentiating hot and cold tumors, and tumor invasion.^[^ ^172^ ^]^ In summary, spatial transcriptomics technology enables the dissection of tumor heterogeneity, and its ongoing developments will reshape the cancer research landscape and open up new possibilities for precise prognosis and treatment of cancer.

## 7 Conclusion and Future Perspectives

Here, we have provided a review of the recent technical aspects of spatial transcriptomics and related fields of application. Spatial transcriptomic technologies are rapidly evolving. This review provides historical background and practical examples in both imaging‐ and NGS‐based spatial transcriptomics.

Using spatial transcriptomics is challenging and the scale of studies should be carefully considered. This sample scale is directly connected to the method of choice, and one may still struggle to use imaging‐based techniques for whole‐brain slices. Instead, gross expression patterns at a voxel of 10–20 µm are practically available on NGS‐based commercial platforms. To reduce experimental cost and improve single‐cell clustering quality, samples can be split into parts, one for non‐spatial regular single‐cell RNA‐seq and the other for the spatial version of the technique. Computational analysis is key to investigating spatial information, and established commercial platforms with modification will soon be available for ease of use.

Spatial technologies are bringing new horizons in investigating epigenomes, chromosome accessibility, chromosomal structures, proteomes, CRISPR gene perturbation, lipid nanoparticles for gene therapy and mRNA vaccine development, AAV serotype optimization, and T‐cell receptor (TCR) sequencing.

Recent reports have demonstrated the spatial version of assay for transposase‐accessible chromatin using sequencing (ATAC‐seq).^[^ ^173^, ^174^, ^175^ ^]^ By combining RNA expression profiling, spatial investigation of chromosome accessibility can precisely define cell types in neuroscience and cancer research. Updated protocols have been reported for cleavage under targets and tagmentation (CUT&Tag) methods optimized for investigating specific histone mark distribution, and such methods for the spatial version are expected to be multiplexed.^[^ ^176^, ^177^, ^178^ ^]^ Epigenomic MERFISH also achieved spatial analysis of single‐cell level epigenetic features by in situ tagmentation followed by antibody‐driven capturing of epigenetic features.^[^ ^179^ ^]^

Cellular indexing of transcriptomes and epitopes (CITE‐seq)^[^ ^180^ ^]^ and RNA expression and protein sequencing (REAP‐seq)^[^ ^181^ ^]^ showed that it is possible to simultaneously analyze single‐cell transcriptome and proteome by employing DNA‐barcoded antibodies. NGS‐based spatial transcriptomics takes this a step further by incorporating antibody‐derived tags during the library generation, allowing for the analysis of spatial multi‐omics data. Spatial multiomics methods such as CITE‐seq with ST,^[^ ^182^ ^]^ 10X Visium,^[^ ^183^ ^]^ and DBiT‐seq^[^ ^184^ ^]^ have facilitated the sophisticated classification of cell types by integrating transcriptome and proteome data, yielding insights into the spatial organization of these molecules in tissue.

Advances in clustered regularly interspaced short palindromic repeats (CRISPR) technology have provided versatile tools for genome editing and gene therapy.^[^ ^185^ ^]^ Genome‐wide CRISPR knockout screens to cell models have helped identify important genes in pathogenic pathways.^[^ ^186^, ^187^ ^]^Pooled CRISPR‐based screen with single‐cell RNA‐sequencing readouts (Perturb‐seq)^[^ ^188^, ^189^, ^190^ ^]^ has been developed by combining CRISPR screening with single‐cell sequencing methods and enables systematic investigation of the knock‐out effect of individual genes at the single‐cell level. Additionally, Perturb‐seq can explore high‐throughput intracellular and cell‐extrinsic spatial features at the single‐cell level.^[^ ^191^ ^]^ The recent spatial version of Perturb‐seq, Perturb‐map, utilizes combinatorial protein barcoding for a detailed spatial context of interactions between the “CRISPR‐perturbed” and surrounding cells. These toolkits will facilitate research on genomic and functional aspects of model organisms and organs with spatial molecular information on cell–cell interactions.

Lipid nanoparticles (LNPs) are evolving for improved RNA‐based therapeutics^[^ ^192^ ^]^ and gene‐editing tools for delivery.^[^ ^193^ ^]^ Barcoded LNPs have been utilized to assess heterogeneity in cellular expression and its effect on LNP‐mediated mRNA delivery.^[^ ^194^ ^]^ The spatial version of this technology may open a new era for gene therapy and its efficacy assay with massive data to better understand the process of LNP formulation and mRNA inserts.

AAV virus serotypes have been actively optimized for target‐specific gene therapy.^[^ ^195^, ^196^ ^]^ Organ and cell‐type specificities have been optimized by screening capsid proteins of AAV serotypes at single‐cell resolution.^[^ ^197^, ^198^ ^]^ Spatial and temporal information acquired from spatial transcriptomics of optimized candidates with AAV serotype barcodes can be employed, and by reading these barcodes from each tissue and cell type, the efficiency of AAV infection can be determined in detail, which should be useful in further optimization.

Spatial transcriptomics and TCR sequencing are essential for tracing and dissecting T‐cell infiltration and their interactions in metastatic cancer tissues.^[^ ^45^, ^199^ ^]^ A recent product from 10X Genomics allows simultaneous investigation of mRNA and TCR information from H&E‐stained tissue slides.^[^ ^200^ ^]^ High resolution and coverage are crucial in understanding the tumor microenvironments and metastases; therefore, the sensitivity and coverage of sequences need further improvement.

For clinical applications, spatial transcriptomics should be effective in clinical practice and sampling environments. Nucleic acids in human tissue specimens are readily degraded prior to cryopreservation. Certain clinical institutions, such as university hospitals, have limited resources for deep freezing to preserve nucleic acids from nuclease digestion. Moreover, histological diagnosis and research are based on FFPEsamples; therefore, next‐generation technologies should be effective in analyzing heavily fixed samples. 10X Genomics has recently announced two platforms for NGS‐based and imaging‐based spatial transcriptomes for FFPE samples.^[^ ^201^ ^]^ NanoString Technologies reported the compatibility of their CosMx Spatial Molecular Imager (SMI) platform with FFPE samples.^[^ ^202^ ^]^ Based on these commercial platforms, spatial transcriptomics can be more easily applied in clinical research.

Finally, spatial information obtained from clinical samples should yield new classes of biomarkers that are often underestimated because of the lack of appropriate investigative technologies. Therefore, seamlessly integrating multiple sources of transcriptome data that may or may not include spatial or molecular information is important. Computational efforts to analyze massive spatial transcriptomic data may help improve the success of clinical applications for marker discovery and drug development.

## Conflict of Interest

The authors declare no conflict of interest.

## Author Contributions

S.H.J. and R.H.L. contributed equally to this work. H.‐E.P. and C.H.S. conceptualized the contributions of all technologies. H.‐E.P. and C.H.S. wrote the part for imaging and NGS‐based methods, application notes in neuroscience, and future directions and conclusions. S.H.J. and T.K. wrote the computational analysis part. R.H.L. and J.P. wrote the application notes for cancer studies. C.P.M., C.W.L., and J.K.H. reviewed and revised the manuscript and provided critical comments.
